# Supplementary material for: Fumarate Hydratase Loss Causes Combined Respiratory Chain Defects
Source: Cell Rep. 2017 Oct 24;21(4):1036–47. doi: 10.1016/j.celrep.2017.09.092 (PMC5668630; doi:10.1016/j.celrep.2017.09.092)
Supplement: Document S1. Figures S1–S3 [file mmc1.pdf]

Cell Reports, Volume *21*

Supplemental Information

Fumarate Hydratase Loss Causes Combined Respiratory Chain Defects

Petros A. Tyrakis, Marie E. Yurkovich, Marco Sciacovelli, Evangelia K. Papachristou, Hannah R. Bridges, Edoardo Gaude, Alexander Schreiner, Clive D'Santos, Judy Hirst, Juan Hernandez-Fernaud, Roger Springett, John R. Griffiths, and Christian Frezza

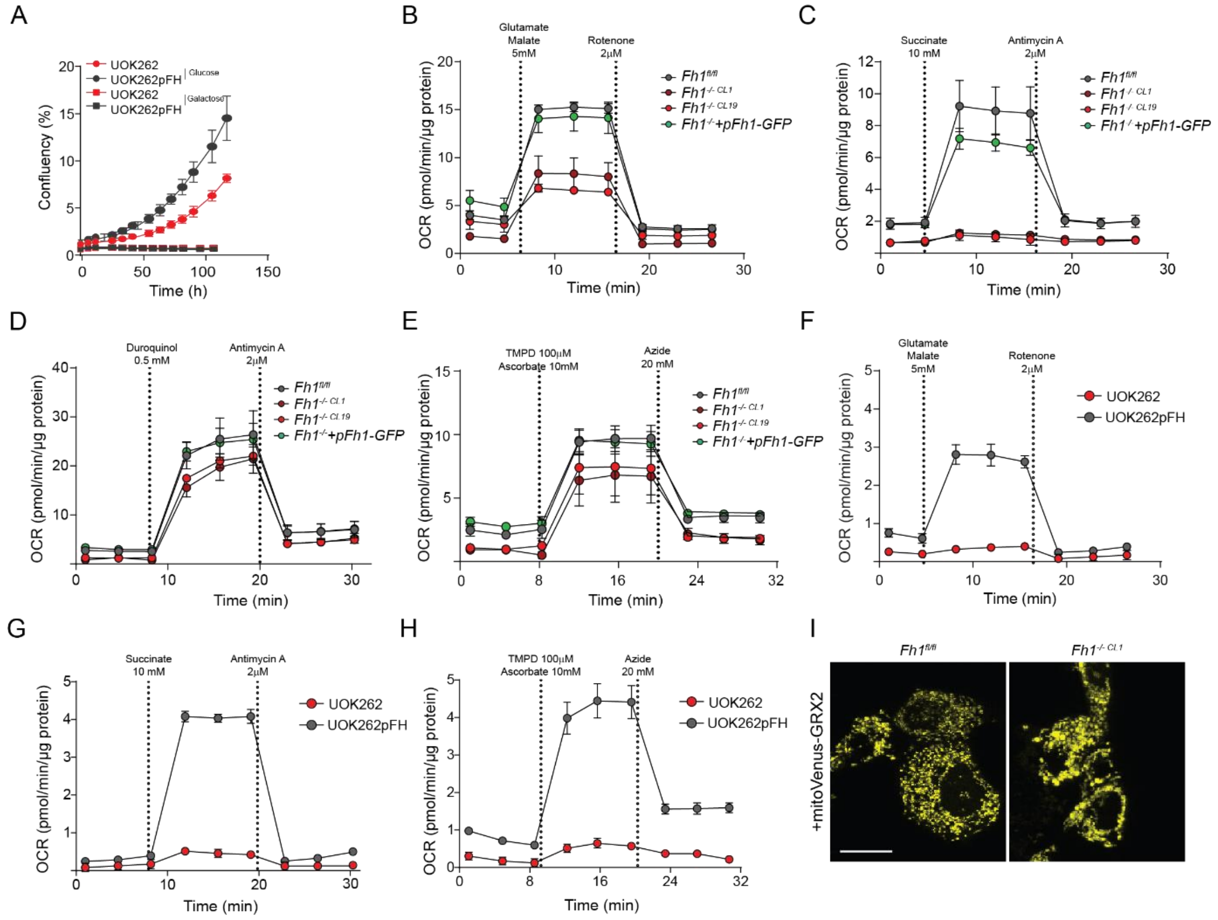

Figure S1: FH-deficient human cells display decreased respiration (related to Figure 1).

(**A**) Growth of indicated cells in 25 mM galactose (squares) or glucose (circles). Data are represented as mean ± SD. (**B-E**) Complete respiration profile of the data presented in Figure 1 and relative to mouse Fh1-deficient cells permeabilized with PMP and in the presence of substrates specific for complex I (**B**), II (**C**), III (**D**) and IV (**E**). Values are normalised for total protein content. **(F-H**) complex I (**F**), complex II (**G**), complex IV (**H**)-driven respiration in the indicated cell lines. Substrates to drive respiration and inhibitors are indicated in the graphs. Concentrations of these compounds are indicated in both the graphs and the methods section. Data were generated from at least 3 independent experiments and presented as average ± SEM**.** For respiration through CIII and CIV cells were pre-treated with Rotenone (2 μM) alone or in combination with Antimycin (2 μM) respectively before the addition of the substrates. Duroquinol was not added to the background wells used for normalisation. (**I**) Representative confocal images of cells transfected with mito-Venus-GRX2. Bar = 11.65 μm

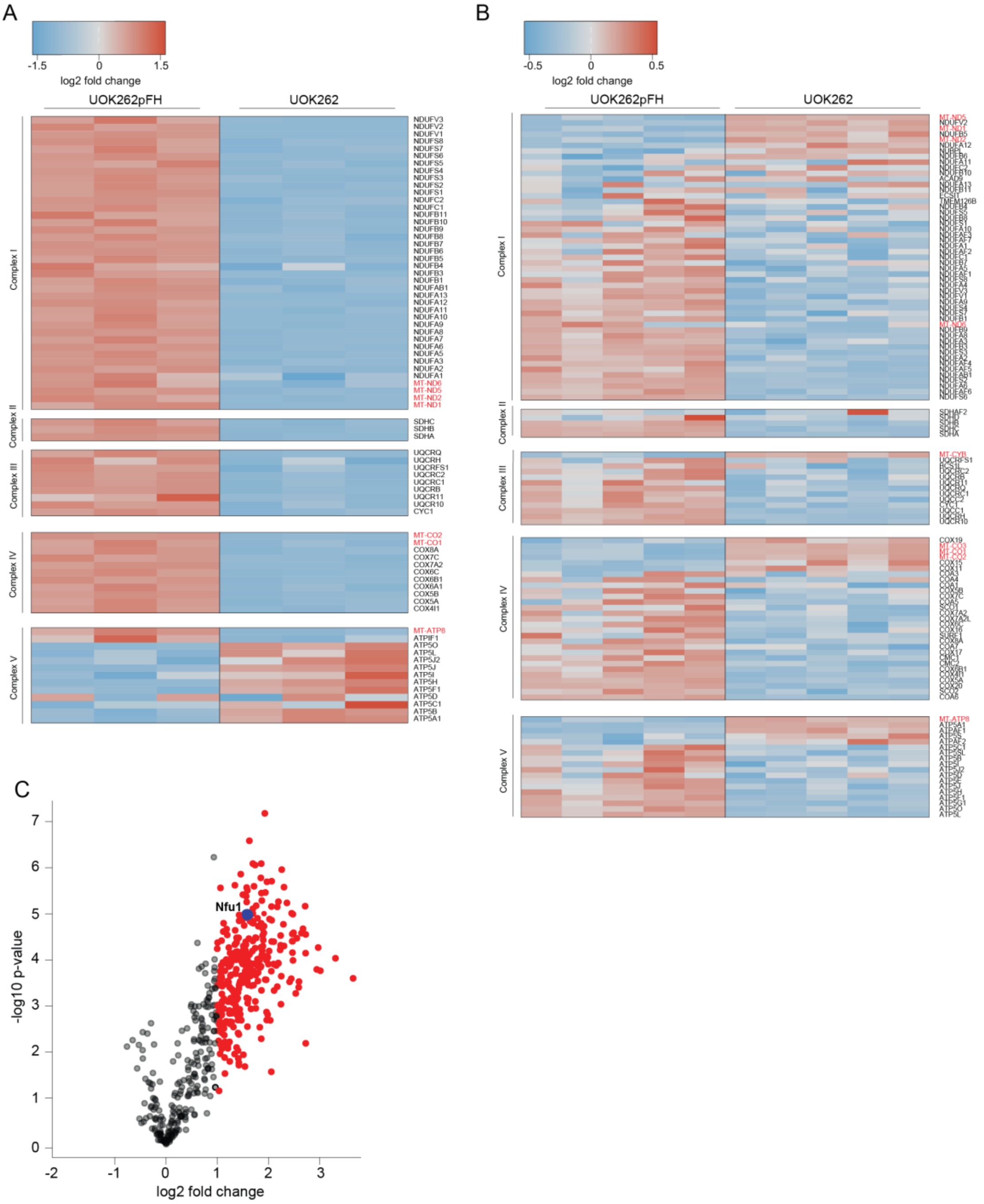

Figure S2: Abundance of respiratory chain complex subunits and assembly factors in FH-deficient cells (related to Figure 3).

(**A**) Heatmap of proteins of respiratory chain complex subunits and assembly factors in UOK262 and UOK262pFH cells. The detected subunits and assembly factors are grouped by complex. The relative quantification of N=3 biological replicates is presented. mtDNA-encoded proteins are indicated in red. (**B**) Heatmap of mRNA levels for all RC subunits from RNA-seq data in UOK262 vs UOK262pFH. mtDNA-encoded genes are indicated in red. (**C**) Volcano plot of the succinated peptides identified in UOK262 cells compared to UOK262pFH. Data were obtained from N=3 biological replicates.

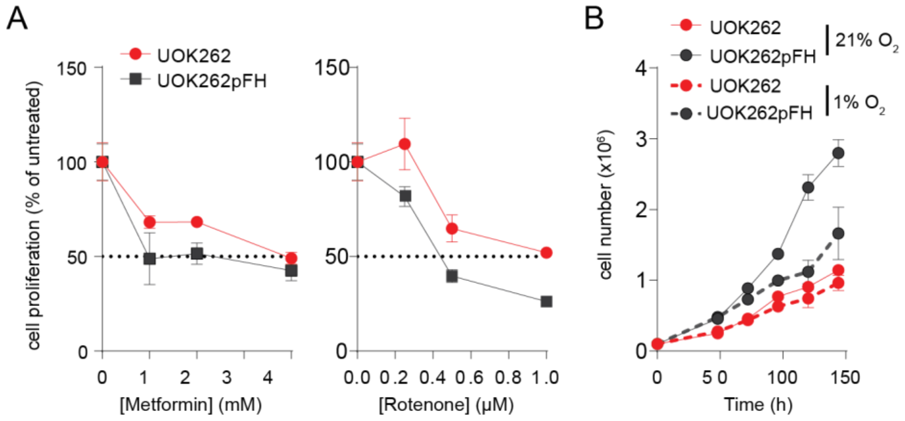

Figure S3: FH-deficient cells are resistant to RC inhibition and hypoxia (related to Figure 5).

(**A-C**) Proliferation rate of the indicated cell lines in the presence of various ETC inhibitors (**A**), and in 21% and 1% oxygen conditions (**B**). Data were obtained from at least 2 independent experiments and are presented as mean ± SD**.**
