# Supplementary material for: Fumarate Hydratase Loss Causes Combined Respiratory Chain Defects
Source: Cell Rep. 2017 Oct 24;21(4):1036–47. doi: 10.1016/j.celrep.2017.09.092 (PMC5668630; doi:10.1016/j.celrep.2017.09.092)
Supplement: Document S2. Article plus Supplemental Information [file mmc2.pdf]

Cell Reports

Article

Fumarate Hydratase Loss Causes Combined Respiratory Chain Defects

Graphical Abstract

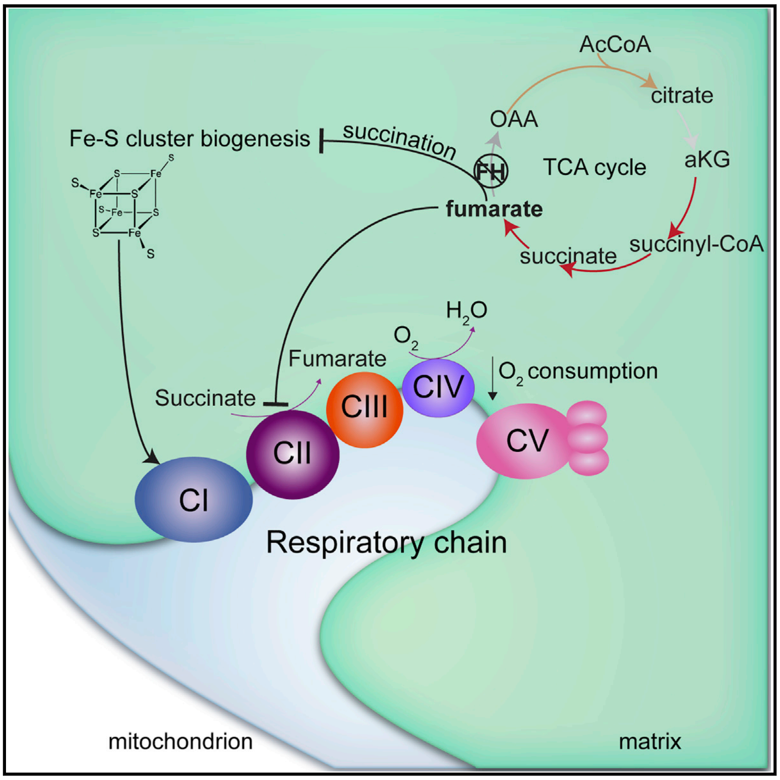

Authors

Petros A. Tyrakis, Marie E. Yurkovich, Marco Sciacovelli, ..., Roger Springett, John R. Griffiths, Christian Frezza

Correspondence

cf366@MRC-CU.cam.ac.uk

In Brief

Tyrakis et al. show that fumarate accumulation in fumarate hydratase-deficient cells leads to the inhibition of respiratory chain (RC) complex II and the succination of proteins involved in the biogenesis of Fe-S clusters required for RC complex I function, enabling these cells to resist insults that target mitochondria.

Highlights

- FH-deficient cells exhibit combined respiratory chain (RC) defects
- Fumarate impairs RC complex II function by product inhibition
- Fumarate causes succination of Fe-S cluster biogenesis proteins
- FH-deficient cells resist unfavorable metabolic conditions, including hypoxia

Tyrakis et al., 2017, Cell Reports *21*, 1036–1047
October 24, 2017 © 2017 The Author(s).
https://doi.org/10.1016/j.celrep.2017.09.092

CellPress

OPEN ACCESS
CellPress

Cell Reports
Article

Fumarate Hydratase Loss Causes Combined Respiratory Chain Defects

Petros A. Tyrakis,[1,8] Marie E. Yurkovich,[2,8] Marco Sciacovelli,[3,8] Evangelia K. Papachristou,[4] Hannah R. Bridges,[5] Edoardo Gaude,[3] Alexander Schreiner,[6] Clive D'Santos,[4] Judy Hirst,[5] Juan Hernandez-Fernaud,[7] Roger Springett,[5,9] John R. Griffiths,[1] and Christian Frezza[3,10,*]

[1]Cancer Research UK Cambridge Institute, University of Cambridge, Robinson Way, Cambridge CB2 0RE, UK
[2]Department of Biochemistry, University of Cambridge, Sanger Building, 80 Tennis Court Road, Cambridge CB2 1GA, UK
[3]Medical Research Council Cancer Unit, University of Cambridge, Cambridge Biomedical Campus, Box 197, Cambridge CB2 0XZ, UK
[4]Proteomics Core Facility, Cancer Research UK Cambridge Institute, University of Cambridge, Robinson Way, Cambridge CB2 0RE, UK
[5]Medical Research Council Mitochondrial Biology Unit, University of Cambridge, Wellcome Trust/MRC Building, Hills Road, Cambridge CB2 0XY, UK
[6]PerkinElmer, Inc., Schnackenburgalle 114, 22525 Hamburg, Germany
[7]School of Life Sciences, Gibbet Hill Campus, University of Warwick, Coventry CV4 7AL, UK
[8]These authors contributed equally
[9]Present address: BHF Centre of Research Excellence, King's College London, The James Black Centre, 125 Coldharbour Lane, London SE5 9NU, UK
[10]Lead Contact
*Correspondence: cf366@MRC-CU.cam.ac.uk
https://doi.org/10.1016/j.celrep.2017.09.092

SUMMARY

Fumarate hydratase (FH) is an enzyme of the tricarboxylic acid (TCA) cycle mutated in hereditary and sporadic cancers. Despite recent advances in understanding its role in tumorigenesis, the effects of FH loss on mitochondrial metabolism are still unclear. Here, we used mouse and human cell lines to assess mitochondrial function of FH-deficient cells. We found that human and mouse FH-deficient cells exhibit decreased respiration, accompanied by a varying degree of dysfunction of respiratory chain (RC) complex I and II. Moreover, we show that fumarate induces succination of key components of the iron-sulfur cluster biogenesis family of proteins, leading to defects in the biogenesis of iron-sulfur clusters that affect complex I function. We also demonstrate that suppression of complex II activity is caused by product inhibition due to fumarate accumulation. Overall, our work provides evidence that the loss of a single TCA cycle enzyme is sufficient to cause combined RC activity dysfunction.

INTRODUCTION

The tricarboxylic acid (TCA) cycle enzyme fumarate hydratase (FH) catalyzes the reversible hydration of fumarate to malate. While homozygous mutations of *FH* leads to fumaric aciduria (OMIM: 606812), a lethal metabolic disorder, its heterozygous mutations cause hereditary leiomyomatosis and renal cell cancer (HLRCC), a cancer syndrome characterized by uterine fibroids, cutaneous leiomyoma, and type 2 papillary renal cell cancer (Tomlinson et al., 2002). The oncogenic properties of *FH* loss have been mostly ascribed to the intracellular accumulation of fumarate. High levels of this metabolite inhibit hypoxia inducible factors (HIF) prolyl hydroxylases, leading to HIF stabilization (Isaacs et al., 2005). Furthermore, fumarate causes the non-enzymatic covalent modification of reactive cysteine residues in proteins, a process known as succination (Alderson et al., 2006). This novel post-translation modification can alter protein function (Ternette et al., 2013), and it has been suggested to inactivate kelch-like ECH associated protein 1 (KEAP1), leading to the upregulation of an antioxidant response mediated by the transcription factor nuclear factor, erythroid 2 like 2 (NFE2L2) (Adam et al., 2011; Ooi et al., 2011). We also demonstrated that fumarate leads to the epigenetic suppression of a family of anti-metastatic microRNAs, *MIR200*, leading to an epithelial-to-mesenchymal transition, a phenotypic switch that promotes tumor initiation and metastasis (Sciacovelli et al., 2016).

Because of its role as key enzyme of the TCA cycle, FH deficiency leads to profound metabolic changes, including decreased mitochondrial respiration (Frezza et al., 2011; Yang et al., 2010, 2012), alteration of TCA cycle functionality (Frezza et al., 2011), and reversal of the urea cycle (Zheng et al., 2013). However, the interplay between loss of FH, increased fumarate levels, and different aspects of mitochondrial biology is still unclear. Here, we investigate the effects of FH deficiency on mitochondrial function and show that FH loss leads to a complex dysregulation of the respiratory chain (RC), by affecting multiple RC complexes. Our results uncover unexpected bioenergetic features of FH-deficient cells that could explain their ability to survive under low oxygen, a condition experienced by areas of most solid tumors.

RESULTS

Loss of FH Leads to Respiratory Dysfunction

We began this investigation by assessing the respiratory phenotype of mouse and human FH-deficient cells. Mouse epithelial

This is an open access article under the CC BY license (http://creativecommons.org/licenses/by/4.0/).

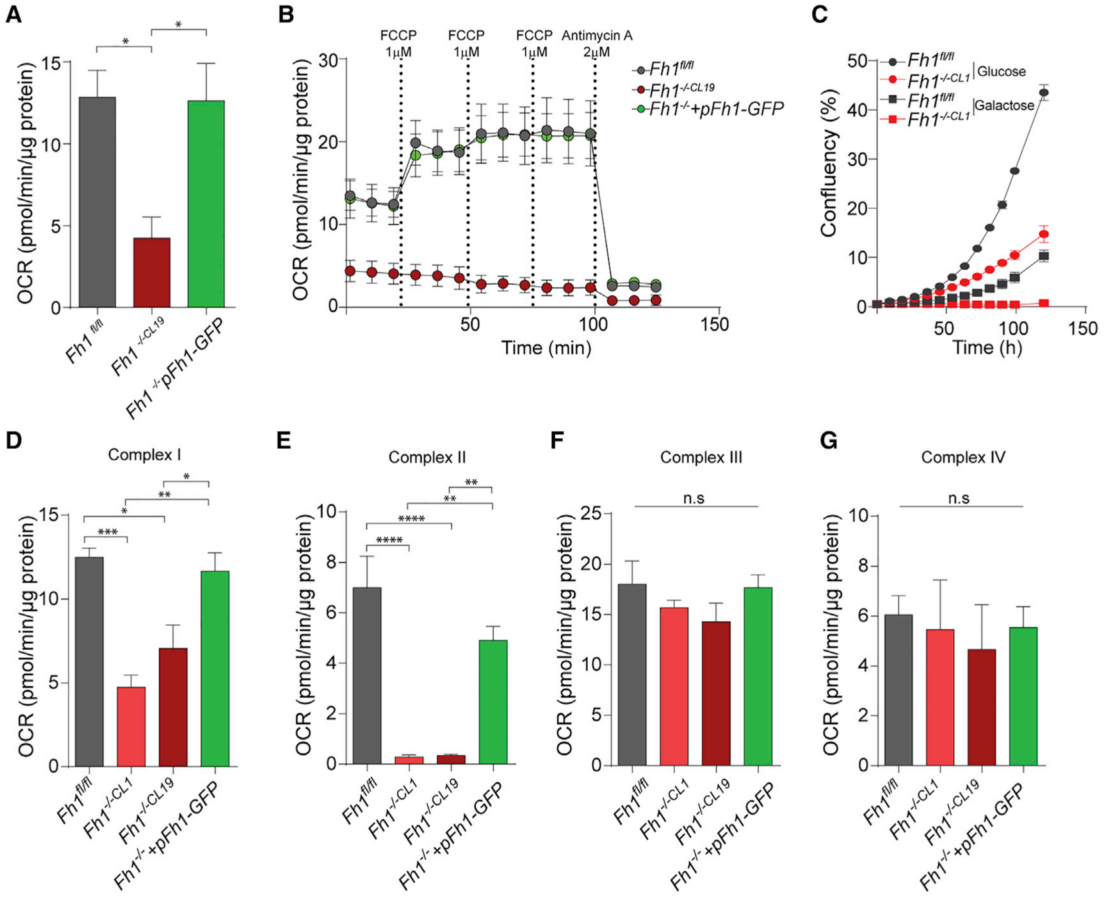

Figure 1. Fh1-Deficient Cells Display Defects in RC Complexes I and II

(A) Basal respiration in the indicated mouse cells. Data are normalized for total protein content and displayed as mean ± SEM. One-way ANOVA test was applied to assess the difference in the groups.

(B) Oxygen consumption rate (OCR) in response to increasing concentrations of FCCP followed by antimycin. Data are normalized for total protein content and displayed as mean ± SEM.

(C) Cell growth of indicated cell lines in 25 mM galactose (squares) or glucose (circles). Data are represented as mean ± SD.

(D–G) Complex I- (D), complex II- (E), complex III- (F), and complex IV- (G) driven respiration in the indicated cell lines after subtraction of inhibitor-insensitive respiration values measured after the addition of substrates. Substrates to drive respiration and concentrations of drugs used are indicated in Figure S1. Data were generated from at least 3 independent experiments and presented as mean ± SEM. One-way ANOVA test was applied to assess the difference in the groups. n.s., not significant.

kidney cells were obtained from *Fh1* conditional knockout animals and included Fh1-proficient cells (*Fh1*$^{fl/fl}$) and two Fh1-deficient clones, *Fh1*$^{-/-CL1}$ and *Fh1*$^{-/-CL19}$ (Frezza et al., 2011). An Fh1-reconstituted cell line (*Fh1*$^{-/-}$*+pFh1-GFP*) was generated to determine the effects of restoration of Fh1 activity on an Fh1-deficient background (Sciacovelli et al., 2016). Human FH-deficient cell lines included UOK262 (Yang et al., 2010) and a previously generated FH-reconstituted isogenic cell line, UOK262pFH (Frezza et al., 2011). In accordance with previous work (Frezza et al., 2011), Fh1-deficient cells exhibited a decreased basal respiration (Figure 1A). Furthermore, the protonophore carbonyl cyanide-4-(trifluoromethoxy)phenylhydrazone (FCCP) failed to increase respiration of Fh1-deficient cells, indicating a defect in maximal respiratory capacity (Figure 1B). Importantly, complementation of Fh1 restored normal levels of basal and maximal respiratory capacity (Figures 1A and 1B), indicating that the respiratory defects are reversible and depend on Fh1.

The extent of mitochondrial dysfunction was assessed further by growing cells in a glucose-deficient medium supplemented with galactose, the catabolism of which enhances oxidative metabolism. Mouse and human FH-deficient cells could not grow in the glucose-free galactose-supplemented medium (Figures 1C and S1A), suggesting that they are unable to further increase mitochondrial function for energy generation. The expression of FH in UOK262 cells did not restore their capacity to grow in galactose (Figure S1A), likely because they maintain high rates of glycolysis despite restoration of their mitochondrial function (Frezza et al., 2011). Overall, these results indicate that the loss of FH causes a profound mitochondrial dysfunction.

We then investigated the mechanisms behind the respiratory defects in FH-deficient cells. To this end, we measured cell

respiration upon cell permeabilization with recombinant cytolysin protein (PMP), which selectively permeabilizes the plasma membrane, but not the mitochondrial membranes (Salabei et al., 2014), enabling the determination of the activity of individual RC components. In Fh1-deficient mouse cells, RC complex I activity was reduced to about 30% of that of *Fh1*-proficient cells (Figures 1D and S1B). In addition, Fh1-deficient mouse cells displayed a loss of complex II activity (Figures 1E and S1C), whereas the activity of RC complexes III and IV was not significantly altered (Figures 1F, 1G, S1D, and S1E). Compared to mouse Fh1-deficient cells, UOK262 cells displayed a more pronounced respiratory defect. Indeed, alongside defective RC complex I and complex II (Figures S1F and S1G), UOK262 cells exhibited diminished complex IV activity compared to the FH-reconstituted cell line (Figure S1H). In summary, these data indicate that human and mouse FH-deficient cells exhibit defects in RC complexes I and II and that the human cells also exhibit defective RC complex IV.

High Levels of Fumarate Inhibit Complex II but Not Complex I

The observation that respiration is impaired in PMP-permeabilized Fh1-deficient cells, in which mitochondria are intact but exposed to exogenous substrates, suggested an intrinsic dysregulation of RC complexes. Importantly, an in-gel flavin-site complex I activity assay performed on blue native gels (Figure 2A) confirmed intrinsic defects in complex I, whose activity was reduced to 30% in Fh1-deficient cells. We then tested whether fumarate, which accumulates at very high levels in these cells, affects complex I activity. To this aim, isolated mitochondrial membranes from bovine heart mitochondria were incubated with increasing doses of fumarate. Neither fumarate nor its cell-permeable derivative monomethyl-fumarate (MMF) affected complex I-mediated respiration, even at high concentration (Figure 2B). These results confirmed the defects in complex I in Fh1-defcient cells but excluded a direct effect of fumarate on this RC complex.

We also investigated the effects of fumarate on complex II. To this end, we measured the succinate-ubiquinone oxidoreductase (SQR) activity of complex II in cells incubated with a detergent that solubilizes the mitochondrial membranes, releasing matrix fumarate. Surprisingly, we found that complex II activity is not decreased in this assay (Figures 2C), in which fumarate levels are in the low micromolar range in Fh1-deficient cells (Figure 2D). Fumarate and succinate bind with similar affinity to the succinate-binding site of succinate dehydrogenase (SDH) A (Grivennikova et al., 1993). Hence, because the intracellular concentration of fumarate in our Fh1-deficient mouse cells is approximately 10 mM, 10-fold higher than succinate (Frezza et al., 2011), it is likely that the concentration of fumarate in the mitochondrial matrix is similarly elevated and that fumarate blocks the access of succinate to the SDHA active site. Indeed, we found that SQR activity is markedly decreased by fumarate in a concentration-dependent fashion (Figure 2E). This inhibition of SDH by fumarate is consistent with a mixed inhibition model (Figures 2F and 2G), supporting the hypothesis that accumulation of fumarate decreases SDH-driven respiration in Fh1-deficient cells by competing with succinate. In summary, these results indicate that, while fumarate has no direct inhibitory effects on complex I, it suppresses complex II activity by product inhibition.

RC Protein Abundance in FH-Deficient Cells

To investigate whether changes in RC activity were associated with specific depletion of RC complexes, we performed tandem mass tagging (TMT) quantitative proteomics (McAlister et al., 2012; Thompson et al., 2003). Overall, we identified and quantified 87% and 92% of the known RC proteins and assembly factors in human and mouse cell lines, respectively. The expression levels of 40 of the 45 complex I subunits were significantly reduced in UOK262 cells compared to UOK262pFH cells (Figure S2A). Furthermore, complex II, complex III, and complex IV showed decreases in abundance of specific subunits (Figure S2A). These data suggest that respiratory dysfunction in UOK262 cells is likely the product of a global downregulation in the content of RC complexes and their assembly factors. To investigate whether the downregulation of RC complexes occurred at a transcriptional level, we analyzed mRNA levels of RC transcripts using a previously published dataset (Sciacovelli et al., 2016). We observed a global transcriptional downregulation of RC subunits in UOK262 compared to UOK262pFH (Figure S2B). Interestingly, mtDNA-encoded RC subunits appeared upregulated in UOK262 (Figure S2B), indicative of a possible compensatory increase in mitochondrial biomass due to the overall suppression of nuclear-encoded RC subunits.

To our surprise, there were no significantly depleted RC proteins in Fh1-deficient mouse cells (Figure 3A). Interestingly, SDH subunits showed increased expression over control, despite the fact that complex II makes no contribution toward respiration in Fh1-deficient cells (Figure 1C). Optical measurement of the functional content of complex III, cytochrome *c*, and complex IV (Kim et al., 2012; Ripple et al., 2013) confirmed that the content of complex III and cytochrome *c* does not change, and a 40% decrease in complex IV was observed (Figure 3B), although the latter is not reflected either by respiratory defects (Figure 1G) or by protein abundance (Figure 3A). These data suggest that the respiratory dysfunction in Fh1-deficient mouse cells cannot be explained by changes in RC complexes' abundance and could be ascribed to protein regulation at the post-translational level. Therefore, we decided to investigate whether post-translational modifications could explain the observed profound respiratory defects in mouse Fh1-deficient cells.

Succination Profile of Mitochondrial Protein in FH-Deficient Cells

Fumarate accumulation is responsible for a post-translation modification termed *succination*, which involves covalent bonding of fumarate to reactive cysteine residues of proteins (Alderson et al., 2006; Bardella et al., 2011; Ternette et al., 2013; Yang et al., 2014). We therefore studied the role of protein succination in the regulation of the activity of RC complexes. To this end, we applied a relative quantitative proteomic experiment using TMT. This non-targeted approach identified 563 (442 significantly changed, false discovery rate [FDR] < 5%) succinated peptides in mouse cells (Figure 4A). Importantly, global succination decreased significantly after the restoration of Fh1 expression (Figure 4B), in line with the reduction in fumarate

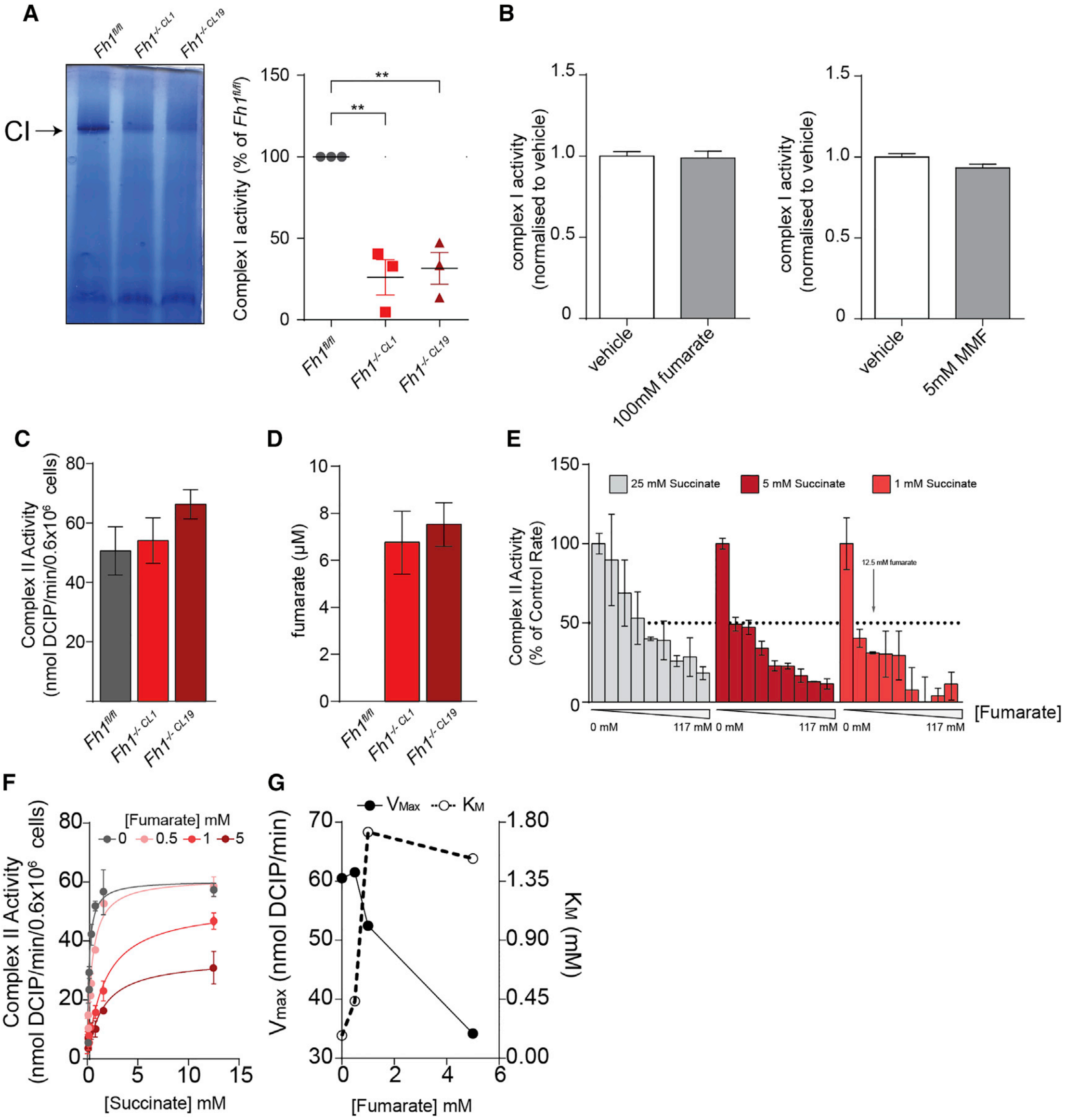

Figure 2. Fumarate Inhibits Complex II, but Not Complex I, Activity

(A) Representative blue native in-gel complex I activity assay in the indicated cell lines. Data were obtained from 3 independent experiments and are presented as mean ± SEM. One-way ANOVA test was applied using Dunnett's multiple comparison test.

(B) Complex I activity in mitochondrial membranes from bovine heart in the presence of the indicated concentration of fumarate and monomethyl-fumarate (MMF). Data are normalized to vehicle-treated condition and presented as mean ± SD from 4 replicates.

(C) Succinate-Quinol Reductase (SQR) activity of complex II in the indicated cell lines. Values are presented as mean ± SEM.

(D) Concentration of fumarate in the SQR assays showed in (C). Values are presented as mean ± SEM.

(E) SQR activity of complex II in the presence of the indicated concentrations of succinate and increasing concentration of fumarate. Values are presented as mean ± SD.

(F) Michealis-Menten representation of SQR activity of complex II in the presence of different concentrations of fumarate and succinate. Values are presented as mean ± SD.

(G) Calculated V_{max} of complex II and K_M of complex II for succinate, as a function of fumarate concentration. Data were obtained from 2 independent experiments.

levels, and restored mitochondrial respiration observed in Fh1-reconstituted cells. While no RC subunits were identified in the succinated proteome, we identified succination of multiple components of the iron-sulfur (Fe-S) cluster biogenesis family of proteins. Succinated residues were detected on cysteine 213 of the Fe-S Cluster Scaffold Nfu1, on two cysteine residues (Cys-70 and Cys-139) of the Fe-S cluster assembly enzyme (Iscu), on cysteine 216 of bolA family member 1 (Bola1), and on

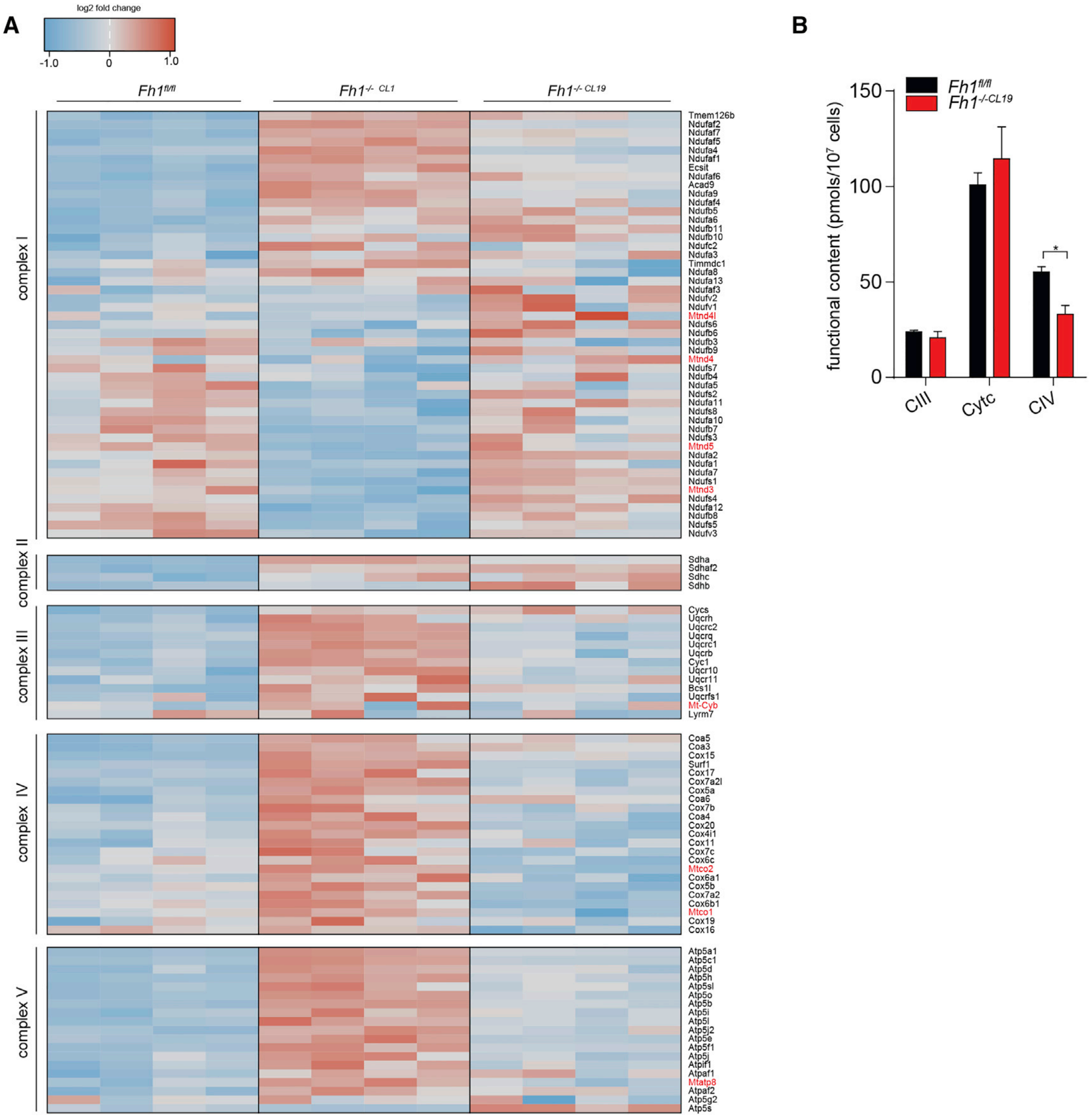

Figure 3. Abundance of RC Complex Subunits and Assembly Factors in Fh1-Deficient Mouse Cells

(A) Quantitative profile of RC complex subunits and assembly factors in the indicated cell lines. The detected subunits and assembly factors are grouped by RC complexes. mtDNA-encoded subunits are indicated in red. The combined data of 4 biological replicates run over two independent TMT analyses are presented.

(B) Functional content of complex III, cytochrome *c*, and complex IV in the indicated cell lines. Values are represented as mean ± SEM. Unpaired t test was used for statistics.

cysteine 110 of Bola3 in Fh1-deficient cells (Figure 4A). Of note, none of these proteins exhibited significant alterations in their protein levels (Figure 4C). We then focused on Nfu1, which was the most significantly succinated protein among the ones involved in Fe-S cluster biogenesis (Figure 4A). Importantly, Fh1 re-expression significantly decreased Nfu1 succination in *Fh1*$^{-/-}$*+pFh1-GFP* cells (Figure 4D) to a level comparable to *Fh1*$^{fl/fl}$ (Figures 4D and 4F). To confirm the link between fumarate accumulation and succination of Fe-S cluster proteins, we performed a similar proteomic analysis in human cells. Here we detected 862 (639 significantly changed, FDR < 5%) peptides in human FH-deficient cells (Figure S2C). Strikingly, we identified NFU1 as one of the succinated proteins in UOK262 cells, and its levels of succination were rescued upon FH expression in UOK262pFH (Figure S2C).

The cysteine residues of Iscu and Nfu1 affected by succination are critical for their enzymatic activity. Consistently, mutations in these enzymes lead to defects in the early steps of Fe-S biosynthesis and varying degrees of deficiency of the RC complexes (Navarro-Sastre et al., 2011; Parent et al., 2015). Therefore, we hypothesized that succination of Iscu/Nfu1/Bola1-3 would, at least in part, explain the mitochondrial respiratory defects observed in Fh1-deficient cells. Other mitochondrial enzymes, including aconitase 2 (Aco2), require correct Fe-S assembly for

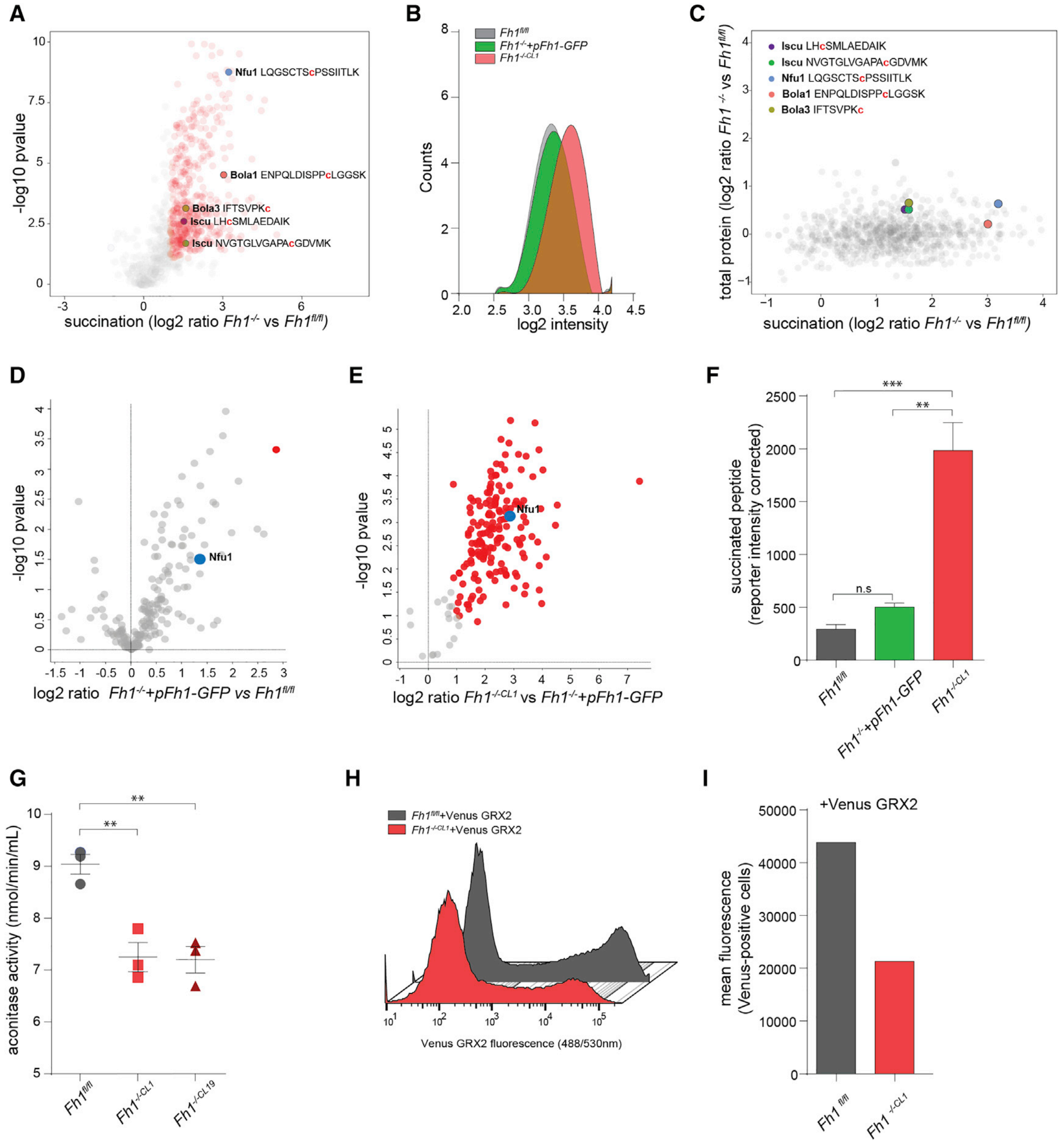

Figure 4. Fumarate-Mediated Succination of Fe-S Cluster Biogenesis Enzymes

(A) Volcano plot illustrating the quantitative profile of identified mouse succinated peptides found in *Fh1*$^{-/-}$ (both knockout [KO] clones) compared to *Fh1*$^{fl/fl}$ cells. Nfu1, Iscu, Bola1, and Bola3 succinated peptides are indicated. Red dots indicate significantly regulated succinated peptides in Fh1-deficient cells compared to Fh1-proficient cells.

(B) Polynomial curve representing the distribution of the intensity of succination signal in mouse cells (x) versus counts (y).

(C) Scatterplot showing the levels of total protein changes compared to changes in succination. The succinated peptides display high fold changes (x axis) whereas the respective total protein levels remain mostly unchanged (y axis). The succinated peptides from Iscu, Nfu1, Bola1, and Bola 3 proteins are indicated.

(D) Volcano plot of succinated peptides in *Fh1*$^{-/-}$*+pFh1-GFP* compared to *Fh1*$^{fl/fl}$.

(E) Volcano plot of succinated peptides in *Fh1*$^{-/-}$ compared to *Fh1*$^{-/-}$*+pFh1-GFP*.

(F) Bar graph indicating the reporter intensity relative to the Nfu1 succinated peptide containing C213 in the indicated cell types. Values are represented as mean ± SEM. One-way ANOVA test followed by Sidak's multiple comparison test was applied to assess the difference in the groups. n.s., not significant.

(G) Aconitase activity in Fh1-deficent cells (3 biological replicates, values represents mean ± SEM). One-way ANOVA test followed by Dunnett's multiple comparison test was applied to assess the difference in the groups.

(H and I) FACS profile (H) and fluorescence intensity of Venus-positive cells (I) of cells of the indicated genotype transfected with mito-Venus-GRX2.

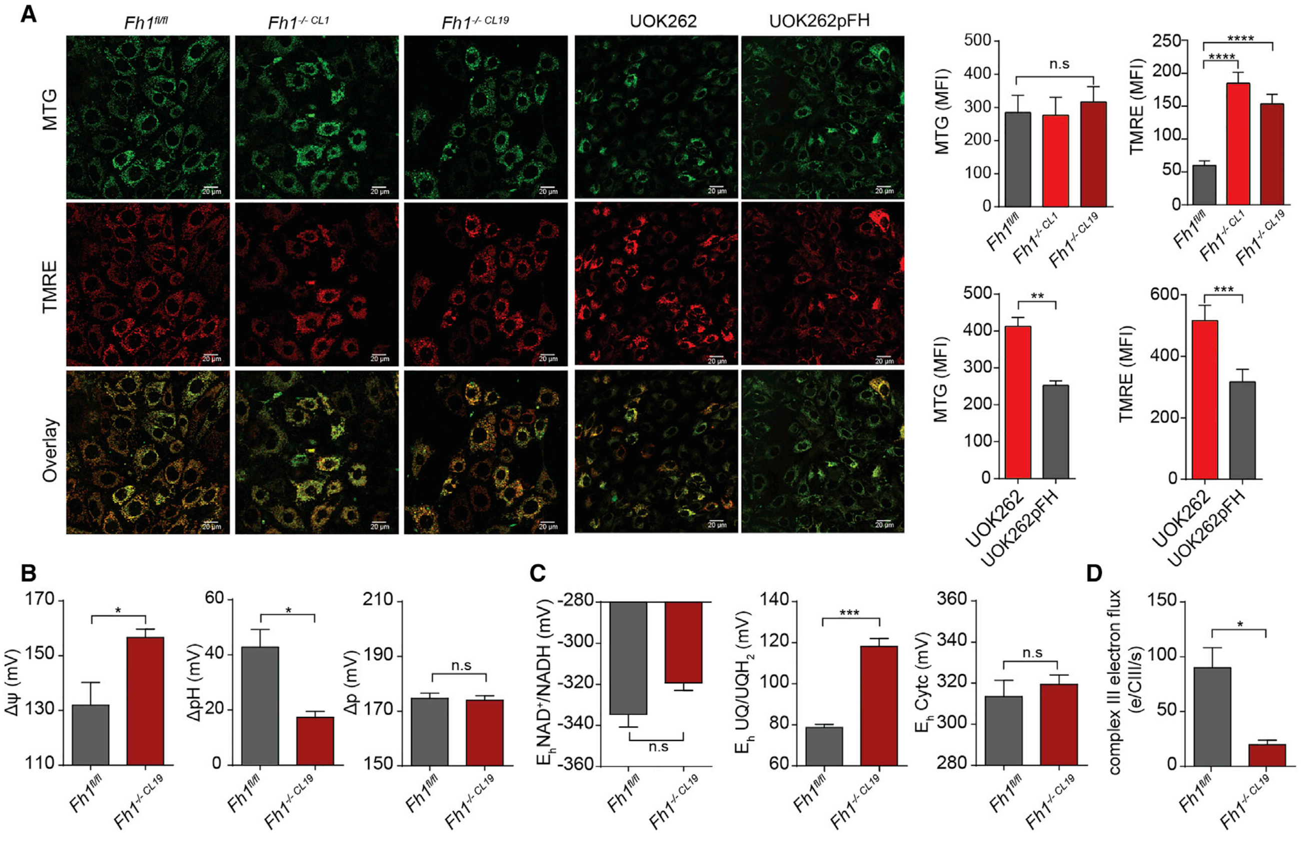

Figure 5. Fh1-Deficient Cells Display Normal ΔP but Altered ΔpH and $\Delta\Psi$

(A) TMRE and MTG staining and quantification in the indicated cell lines. Values are presented as mean ± SEM (MTG) or mean ± SD (TMRE). One-way ANOVA test was applied using Bonferroni multiple comparison test for mouse cells. Unpaired t test was used for UOK262/pFH cells.

(B and C) Multi-wavelength spectrophotometric determination of $\Delta\Psi$, ΔpH, and ΔP (B) and of the redox state of the ubiquinone/ubiquinol couple, cytochrome *c*, and the NAD^+/NADH couple (C). Values are presented as mean ± SEM. Unpaired t test was applied. For the ΔpH graph, 61 mV = 1 pH unit difference.

(D) Respirometry coupled with multi-wavelength spectrophotometric determination of the flux of electrons through complex III. Data were obtained from at least 3 independent cultures and presented as mean ± SEM. Unpaired t test was applied to assess the difference in the groups. n.s., not significant.

their function and can be used as a beacon for Fe-S cluster biogenesis. Therefore, to validate the downstream effect of Fe-S imbalance in our cells lines, we measured the activity of Aco2. Consistently, Aco2 activity was significantly decreased in Fh1-deficient cells (Figure 4G). To further demonstrate a defect in the biogenesis of the Fe-S cluster, we used mito-Venus-GRX2, a well-established probe whose fluorescence depends on the abundance of mitochondrial Fe-S clusters (Hoff et al., 2009). Importantly, mito-Venus-GRX2 fluorescence, which exhibited a typical mitochondrial staining (Figure S1I), was markedly reduced in Fh1-deficient cells (Figures 4H and 4I). Together, these data suggest that succination of the Fe-S cluster promoted by fumarate leads to defects in Fe-S cluster biogenesis, which could explain the defects in RC complex I that we determined in FH-deficient cells.

Mitochondrial Potential Maintenance in FH-Deficient Cells

To investigate whether the observed RC defects have a functional consequence on mitochondrial bioenergetics, we used the potentiometric probe tetramethyl rhodamine ethyl ester (TMRE) to measure the mitochondrial membrane potential. Surprisingly, FH-deficient cells showed a higher accumulation of TMRE in the matrix than their FH-proficient counterparts (Figure 5A), suggesting that they have a higher mitochondrial membrane potential ($\Delta\Psi$) despite having substantial defects in RC activity. To further investigate these findings, we set out to quantify the $\Delta\Psi$ and the pH gradient (ΔpH) across the inner mitochondrial membrane, using multi-wavelength spectrophotometry (Kim et al., 2012; Ripple et al., 2013). This analysis revealed that, while $\Delta\Psi$ is indeed elevated in these cells, in line with our imaging analyses, ΔpH is decreased (Figure 5B) such that the proton motive force (ΔP), which is the sum of the two, remains constant. The decrease in ΔpH is consistent with a decreased matrix pH. Given that the activity of the TCA cycle does not change the pH of the matrix per se, because protons there generated are consumed by the electron transport chain (ETC), it is likely that the high matrix concentration of fumarate and the associated H^+ are major contributors to matrix pH.

The spectrophotometry approach also revealed that, while the redox potentials of the NAD^+/NADH couple and cytochrome *c* are not significantly different, the ubiquinone/ubiquinol couple

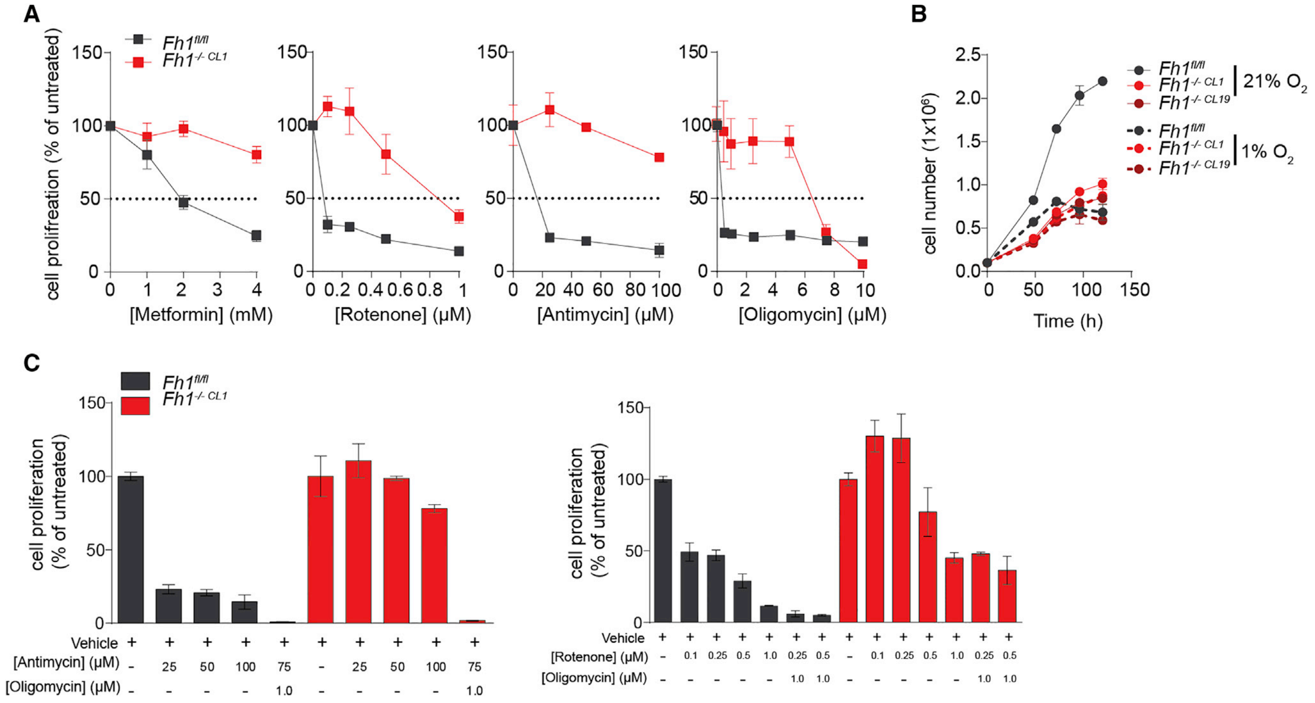

Figure 6. Fh1-Deficient Cells Are Resistant to RC Inhibition and Hypoxia

(A–C) Proliferation profile of the indicated cell lines in the presence of various ETC inhibitors (A), in 21% and 1% oxygen conditions (B), or in response to the indicated mitochondria inhibitors (C). Data were obtained from at least 2 independent cultures and presented as mean ± SD.

is markedly more oxidized in Fh1-deficient cells (Figure 5C), consistent with combined defects for complexes I and II; and it confirmed that the electron flux, measured as the number of electrons transferred per complex III per second, is decreased in Fh1-deficient cells (Figure 5D). Together, these results indicate that the overall flux of electrons through the RC is decreased in Fh1-deficient cells and that there is a different contribution of $\Delta\Psi$ and ΔpH to ΔP.

Finally, to test whether FH-deficient cells are less dependent on mitochondrial function for cell survival, we treated FH-deficient cells with a number of RC inhibitors and measured cell growth. As expected from cells adapted to mitochondrial dysfunction, mouse and human FH-deficient cells were more resistant to RC inhibition (Figures 6A and S3A) and their growth was unimpeded in low-oxygen conditions (Figures 6B and S3B). Of note, Fh1-deficent cells were resistant to metformin and rotenone, confirming that complex I is not completely functional. Interestingly, mouse Fh1-deficient cells were sensitized to RC inhibitors in the presence of oligomycin (Figures 6C), consistent with the fact that they still need some contribution of complex V for ATP generation.

DISCUSSION

There is increasing recognition that alterations in cellular metabolism underlie crucial aspects of oncogenesis. The clearest examples of this are seen from mutations in FH, SDH, and isocitrate dehydrogenase (IDH1/2), which give rise to accumulation of the oncometabolites fumarate, succinate, and 2-hydroxyglutarate, respectively (Baysal et al., 2000; Dang et al., 2009; Niemann and Müller, 2000; Tomlinson et al., 2002). In fact, tumor-associated cellular properties can be elicited by treatment with these oncometabolites in the absence of tumorigenic mutations (Losman et al., 2013; Sciacovelli et al., 2016). Another well-documented and highly prevalent feature of tumor metabolism is the observation that respiratory dysfunction often accompanies the Warburg effect, which is recognized as a pervasive feature of tumors. In this study, we sought to provide a mechanistic link among FH loss, the accumulation of fumarate, and the ensuing mitochondrial dysfunction.

FH loss impairs TCA cycle activity and decreases the rate at which NADH and succinate are generated for use by the RC, decreasing oxygen consumption even if the RC components are functional. Thus, loss of FH activity would be expected to result in a loss of electrons (oxidation) from the mitochondrial NAD^+/NADH and fumarate/succinate pools. Yet, this hypothesis has not been formally tested. Here, we performed a comprehensive bioenergetics analysis of mouse Fh1-deficient cells and observed a modest (≈16 mV) oxidation in the NAD^+/NADH pool (Figure 5C), and oxidation of the fumarate/succinate pool was inferred from the increase in cytosolic fumarate. These cells also maintain the same ΔP as Fh1-proficient cells, that is, there is the same amount of energy available for ATP generation by the ATP synthase in both cell types. Oxygen consumption and ATP production are tightly coupled by the relative stoichiometry of the proton-pumping complexes and the ATP synthase, such that a decrease in oxygen consumption and electron flux implies a decrease in mitochondrial ATP production. As the rate of ATP

production and consumption must also be matched, the results imply either that the Fh1-deficient cells have decreased ATP demand or that the shortfall is made up by increased glycolysis and glycolytic ATP production. The latter scenario is corroborated by previous analyses (Frezza et al., 2011; Yang et al., 2012).

The observation that the oxidation in the UQ/UQH_2 ($\approx$39 mV) is much more substantial (Figure 5C) than the oxidation in the NAD^+/NADH pool is consistent with combined defects with complex I and complex II. Indeed, measurement of RC activity showed that complex I activity is decreased in both human and mouse FH-deficient cells, due to incomplete formation and assembly of the Fe-S clusters because of succination of proteins involved in Fe-S cluster biogenesis, including Iscu, Bola1-3, and Nfu1. Although in human cells we confirmed the succination of NFU1, in these cells we also observed a general suppression of nuclear-encoded mitochondrial genes, which likely contributes the observed respiratory defects. We also demonstrated that complex II activity is lowered by product inhibition due to aberrant fumarate accumulation. Complex II contains three Fe-S clusters; therefore, the observation that its activity is intact, when assessed in cell extracts of Fh1-deficient cells, indicates that the defects in Fe-S cluster generation are modest and affect complex I, which contains the highest number of Fe-S clusters among RC complexes, more strongly than complex II. The observed upregulation of SDH assembly factors could also compensate for the decrease in Fe-S cluster biogenesis, increasing the efficiency of their incorporation in the mature protein.

Several reasons could explain the different mitochondrial profiles of human and mouse cells. First, the human cells express a mutant FH (Yang et al., 2012) whose role and function is not completely understood, while the mouse cells are knocked out for Fh1. Also, the different succination profile of Fe-S cluster biogenesis proteins could be explained by the different levels of accumulation of fumarate in these different cell lines (Zheng et al., 2013). In this scenario, NFU1 seems to be more susceptible to succination than Iscu and Bola1/3. Interestingly, Nfu1 succination was identified in T cells treated with fumarate derivatives (Blewett et al., 2016), confirming its vulnerability to succination. The hypothesis that mouse cells exhibit stronger defects in Fe-S cluster biogenesis is also confirmed by the fact that they exhibit inhibition and succination of Aco2 (Ternette et al., 2013), while human cells appear to have intact aconitase activity, as indicated by their ability to perform reductive carboxylation (Mullen et al., 2011). Although the decrease of Aco2 activity has been previously ascribed to succination of cysteine residues involved in Fe-S binding (Ternette et al., 2013), the availability of these residues for succination is more likely the consequence of an early defect in Fe-S assembly. Finally, additional mutations could be present in UOK262 cells, which could explain the transcriptional suppression of mitochondrial enzymes that we observed. These results are similar to those observed in another HLRCC cell line, UOK268, where a genetic suppression of all RC components was observed (Yang et al., 2012). Therefore, more work is required to fully understand the determinants of this transcriptional reprogramming in these cells.

In conclusion, our comprehensive characterization of mitochondrial function in mouse and human FH-deficient cells showed that the loss of a single TCA cycle enzyme is sufficient to alter RC activity via multiple mechanisms, underlining the complex feedback system that controls mitochondrial function in pathophysiological conditions. Interestingly, the metabolic phenotype of FH-deficient cells can more effectively resist conditions that are otherwise unfavorable for mitochondrial function, including hypoxia, suggesting that these changes might be selected during tumor progression.

EXPERIMENTAL PROCEDURES

Cell Culture

Cells were cultured in media supplemented with 10% heat-inactivated fetal bovine serum (FBS). Mouse cells were cultured in high-glucose DMEM (41966, Gibco) supplemented with uridine at a final concentration of 50 μg/mL (Sigma). UOK262 and UOK262pFH cells were cultured in the same medium as *Fh1* cells but without supplemental uridine. For hypoxia experiments, cells were initially grown in normoxia for 24 hr and then transferred into 1% oxygen in an InViVO2 Hypoxia Workstation 500, for the indicated amount of time.

Cell Counting in 21% and 1% O_2

Equal numbers of cells were seeded in 6-well plates and cultured in either 21% or 1% O_2 conditions. At the indicated time points, cells were trypsinized and counted on a Vi-CELL XR (Beckman Coulter), and viability was assessed by the exclusion of trypan blue dye.

Proliferation on Galactose and ETC Inhibitors

Proliferation of cells in the presence of various ETC inhibitors or glucose-deficient medium supplemented with galactose was monitored using an IncuCyte Zoom instrument (Essen Bioscience).

Multi-wavelength Spectrometry

Heme absorption and NADH fluorescence measurements have been described previously (Ripple et al., 2013). In brief, cells were cultured in T175 flasks in phenol red-free DMEM. The cells were washed twice with PBS, detached with TripLE Express (Themo Fisher Scientific), and then the TrypLE was quenched with DMEM supplemented with 10% FBS. Cells were spun down at 500 × *g* for 5 min and re-suspended in FluoroBrite DMEM (Themo Fisher Scientific) supplemented with 4 mM glutamine at a density of 10^7 cells/mL. Studies were carried out in a stirred 5-mL chamber maintained at 37°C. Oxygen consumption, at constant oxygen tension, was calculated from the rate of the diffusion of oxygen across 80 mm of fine-bore oxygen-permeable silicone tubing immersed in the cell suspension. Optical spectra were collected on two time-multiplexed 0.3-m spectrographs (Triax 320; Horiba, Edison, NJ), each equipped with a 1,024 × 128 pixel back-thinned charge-coupled device camera (DV401BV; Andor Technology, SouthWindsor, CT). NADH fluorescence was excited with a high power 365 nm LED (Nichia, Japan), and spectra were collected between 390 and 661 nm using a 300-g/mm grating blazed at 500 nm with the slits set to a spectral resolution of 10 nm. The cell suspension was illuminated with a high-power cool white LED (Lumileds, USA), and heme absorption spectra were collected between 509 and 640 nm using a 600-g/mm grating blazed at 500 nm with slits set to a spectral resolution of 1 nm.

The contribution of each heme/fluorophore to the spectra was calculated using linear multi-wavelength least-squares fitting. The oxidation state and content of the hemes and the oxidation state of mitochondrial NADH pool were back-calculated from the fully reduced state and the fully oxidized state. The fully reduced state was obtained by making the cells anoxic for 2 min at the beginning of the study, and the mitochondrial NADH pool was fully oxidized by inhibiting the TCA cycle with 1 mM of the complex II inhibitor 3-nitropropionic acid and adding 1 μM carbonyl cyanide m-chlorophenyl hydrazine (CCCP) at the end of the study. The hemes were fully oxidized by the subsequent addition of 1 μM rotenone. The residual oxygen consumption after the addition of rotenone was assumed to be non-mitochondrial and

subtracted from the total oxygen consumption to give the mitochondrial oxygen consumption. The electron flux through complex III was calculated by scaling the mitochondrial oxygen consumption by 4 electrons per O_2 and normalizing to the content of complex III measured from the content of the *b*-hemes. The redox potentials of the mitochondrial NAD^+/NADH pool and cytochrome *c* were calculated from the Nernst equation with midpoint potentials of −320 and + 260 mV, and the former was corrected for the pH of the matrix using the pH gradient assuming the cytosol remained at pH 7.0. The redox potentials of the ubiquinone pool, the membrane potential ($\Delta\psi$), and the pH gradient (ΔpH) were calculated from the redox poise of complex III, as described previously (Kim et al., 2012). The proton motive force (Δp) is the sum of $\Delta\psi$ and ΔpH.

Proteomic Analysis

Sample Dissolution and Protein Digestion

Cell pellets were dissolved in 0.1% SDS and 0.1 M Triethylammonium bicarbonate (TEAB) followed by probe sonication. Protein concentration was measured with the Quick start Bradford assay (Bio-Rad). A total 90 μg protein per sample was reduced with 2 μL 50-mM tris-2-carboxymethyl phosphine (TCEP) (Sigma) at 60°C for 1 hr. Cysteine residues were blocked with 1 μl 200-mM methyl methanethiosulfonate (MMTS) (Sigma) for 10-min incubation at room temperature. For protein digestion, 6 μl trypsin solution (Roche) in 0.1% F.A (500 ng/μL) was added to each sample for overnight proteolysis.

TMT Labeling and bRP Fractionation

TMT10plex labeling reagents (Thermo Scientific) were reconstituted in 41 μL anhydrous acetonitrile and transferred to each sample followed by incubation for 1 hr at room temperature. The reaction was quenched with 8 μL 5% hydroxylamine. The peptide mixture was fractionated with high-pH reversed-phase chromatography on a C18 column (Waters, X-bridge). Mobile phase (A) was composed of 20 mM ammonium hydroxide and mobile phase (B) was composed of 80% acetonitrile and 20 mM ammonium hydroxide. The gradient elution method at a flow rate of 200 μL/min was as follows: for 10 min gradient up to 5% (B), for 35 min gradient up to 35% (B), for 10 min up to 50% (B), for 10 min up to 95% (B), for 10 min isocratic to 95% (B), for 5 min down to 5% (B), and for 10 min isocratic equilibration 5% (B) at 40°C. Fractions were collected in a peak-dependent manner and were dried with a vacuum concentrator.

Liquid Chromatography-Mass Spectrometry Analysis

The peptide fractions were analyzed on a Dionex Ultimate 3000 UHPLC system coupled with Q-Exactive mass spectrometer (Thermo Scientific). The RP fractions were reconstituted in 40 μL loading solution (2% acetonitrile and 0.1% formic acid), and a 4-μL volume was loaded on the Acclaim PepMap 100, 100 μm × 2 cm C18, 5 μm, 100 Å trapping column with the ulPickUp Injection mode using the loading pump at a 4 μL/min flow rate for 10 min. For the peptide separation, the Acclaim PepMap RSLC, 75 μm × 50 cm, nanoViper, C18, 3 μm, 100 Å column retrofitted to an easy source was used for multistep gradient elution. Mobile phase (A) was composed of 2% acetonitrile, 0.1% formic acid, and 5% DMSO and mobile phase (B) was composed of 80% acetonitrile, 0.1% formic acid, and 5% DMSO. The gradient elution method at a flow rate of 300 nL/min was as follows: for 10 min gradient up to 5% (B), for 85 min gradient up to 45% (B), for 5 min up to 95% (B), for 8 min isocratic to 95% (B), for 2 min down to 5% (B), and for 10 min isocratic equilibration 5% (B) at 40°C. The top 10 precursors were selected with Fourier transform (FT) mass resolution of 70,000 and isolated for higher-energy collisional dissociation (HCD) fragmentation with collision energy of 33 and FT resolution of 35,000.

Database Search

The acquired tandem mass spectrometry (MS/MS) mass spectra were processed with SequestHT implemented on the Proteome Discoverer software version 2.1 for peptide and protein identifications against a UniProtKB/Swiss-Prot fasta file containing 21,530 reviewed human entries or 17,000 reviewed mouse entries. The SequestHT node included the following parameters: Precursor Mass Tolerance 20 ppm, Fragment Mass Tolerance 0.02 Da, Dynamic Modifications were Oxidation of M (+15.995 Da), Deamidation of N, Q (+0.984 Da), Methylthio of C (+45.988), and 2-succinyl cysteine (+116.011). The Static Modifications were TMT6plex at any N terminus and K (+229.163 Da). The level of confidence for peptide identifications was estimated with the Percolator node with decoy database search. FDR was set at 0.01 based on q-value. The Reporter Ion Quantifier node included a custom TMT 10plex (Thermo Scientific Instruments) Quantification Method, integration window tolerance of 20 ppm, and the Most Confident Centroid integration method.

Statistical Analysis

Protein quantification was normalized on total peptide amount, and the FH-deficient conditions were compared to the wild-type (WT) FH cells with ANOVA test using the Perseus proteomics data analysis tool (Tyanova et al., 2016). Significant hits were filtered using permutation-based FDR (<1% for total proteome and <5% for succinylated peptides). Volcano plots and scatterplots were drawn in R studio using the ggplot2 package. Heatmaps of proteomics data from mouse and human cells were generated with R studio.

RNA Sequencing Analyses

RNA sequencing (RNA-seq) data of UOK262 and UOK262-pFH cells (GEO: GSE77542) were obtained from Sciacovelli et al. (2016). Data were log normalized using the R package DEseq2, and gene expression data of RC complexes were plotted with heatmap representation.

Respirometry Experiments

All respirometry measurements were performed using an XFe24 Analyzer (Agilent Technologies, formerly Seahorse Bioscience) according to the manufacturer's instructions. For experiments on intact cells, injections of various compounds were performed as indicated in the figures. For experiments on permeabilized cells, cell membranes were permeabilized using the Seahorse XF Plasma Membrane Permeabilizer (PMP) reagent according to the manufacturer's instructions. The contribution of each complex to respiration was determined by injections of various compounds, as described by Salabei et al. (2014). Following all respirometry measurements, cells in each well were disrupted in radioimmunoprecipitation assay (RIPA) buffer and protein was measured using the bicinchoninic acid (BCA) assay (Thermo Fisher Scientific). Oxygen consumption rates from each well were normalized to the protein abundance of that well or to the average of the wells relative to that group.

Complex I Activity Assay

Complex I in-gel flavin-site activity assay was measured as indicated in Wittig et al. (2007) using 100 μg isolated mitochondria separated in NativePAGE 3%–12% Bis-Tris Gel System. Gels were scanned and the intensity of complex I activity was measured using ImageJ. Respiration mediated by complex I on isolated mitochondrial membranes from bovine heart was measured as described in Bridges et al. (2014).

SQR Assay

SQR measurements were performed as described by Selak et al. (2005) with minor modifications. Cells were permeabilized with 0.1% v/v Triton X-100 in a buffer with 25 mM $KHPO_4$ (pH 7.4), 1–25 mM succinate, 50 μM decylubiquinone, 5 μM rotenone, 2 μM antimycin A, and 10 mM NaN_3. Different concentrations of fumarate were also included in the buffer, as indicated in the figures. After a 15-min incubation at room temperature, a baseline absorbance at 600 nm was measured and the reaction started by adding 100 μM 26-dichlorophenolindophenol (DCIP) (ϵ = 21 mM^{-1} cm^{-1}). The change in absorbance was monitored for 2 min before and after the addition of 50 μM 2-thenoyltrifluoroacetone (TTFA), a complex II inhibitor used to confirm reaction specificity. The TTFA-sensitive SQR activity is reported as nmol/min DCIP/0.6 × 10^6 cells. Fumarate levels at the end of SQR experiments were measured by ^{1}H nuclear magnetic resonance (^{1}H NMR) spectroscopy. This was performed with solvent suppression on a 600-MHz Brucker Avance NMR spectrometer. 4,4-dimethyl-4-silapentane-1-sulfonic acid (DSS) was used as an internal standard, and the Electronic Reference to Access In Vivo Concentration (ERETIC) method (Akoka et al., 1999) was used to determine the concentration of DSS in each sample. Processing of NMR spectra included both zero- and first-order phase corrections followed by baseline correction using the Chenomx NMR Suite 7.6. Fumarate was identified, based on chemical shift assignment, also using the Chenomx NMR Suite 7.6. The concentration of

fumarate was determined by normalizing peak area to a known concentration of DSS in each sample.

Flow Cytometry Measurements

6×10^5 cells were plated onto 6-cm dishes. The day after, cells were transfected using Lipofectamine 2000, following the manufacturer's instructions and Fe-S constructs N173-GRX2 and C155-GRX2 as described before (Hoff et al., 2009). After 48 hr, cells were washed in PBS and detached using trypsin. After centrifugation at 1,000 × *g* for 5 min, cells were resuspended in complete medium and analyzed by fluorescence-activated cell sorting (FACS) using an LSR II flow cytometer (BD Biosciences). Venus fluorescence was measured using a 488-nm laser for excitation using 50-mW power; emission was measured using a (488) 530/30 detector. 1×10^5 events were acquired for each sample. Graphs presented were generated using FlowJo software.

Aconitase Activity Assay

The activity of aconitase in Fh1-deficient cells was measured using the Aconitase Assay Kit (Cayman), following the manufacturer's instruction.

Microscopy

Cells were grown in μ-Slide 8 Well vessels (ibidi) in medium without phenol red. Live cells were stained with 50 nM MitoTracker Green (MTG) (Invitrogen) and 10 nM TMRE (Invitrogen) for 1 hr at 37°C in complete media with 25 mM HEPES (pH 7.4). Cells were maintained at 37°C and 5% CO_2 in a stage top incubator (Tokai Hit). Images were acquired using a Leica TCS SP5 (Leica Microsystems, Wetzlar, Germany) equipped with a 20× objective. Acquisition was performed in a sequential scanning mode. MTG was excited using the 488-nm laser and TMRE using the 543-nm laser. MTG emission was collected from 500 to 545 nm and TMRE emission from 580 to 620 nm. TMRE intensity from images was quantified using Columbus Image Data Storage and Analysis System (PerkinElmer). For mito-Venus-GRX2 constructs, 8×10^4 or 1×10^5 cells were plated on Nunc Lab-Tek Chambered Coverglass (1.0 borosilicate glass). The day after, cells were transfected with Lipofectamine 2000 (Invitrogen), following the manufacturer's instructions, and mito-Venus Fe-S sensor constructs N173-GRX2 and C155-GRX2. After 16 hr, medium was replaced with a fresh one and images were acquired using a Leica TCS SP5 (Leica Microsystems, Wetzlar, Germany) equipped with a 63× oil UV objective. The Venus construct was excited using Argon laser (514 nm) as described before (Hoff et al., 2009). Microscope settings were kept constant during the acquisition of all images.

Statistical Analysis

Statistical analyses were performed in GraphPad Prism 6 software. Pairwise comparisons were assessed using an unpaired two-tailed Student's t test. Multiple comparisons were assessed with one-way ANOVA, including Tukey post hoc test for multiple testing, if not indicated otherwise. Error bars are shown as indicated in figure legends and all experiments were performed at least twice.

SUPPLEMENTAL INFORMATION

Supplemental Information includes three figures and can be found with this article online at https://doi.org/10.1016/j.celrep.2017.09.092.

AUTHOR CONTRIBUTIONS

Conceptualization, P.A.T., M.E.Y., M.S., and C.F.; Visualization, P.A.T., M.E.Y., M.S., and C.F.; Methodology, P.A.T., M.E.Y., M.S., A.S., H.R.B., J.H., J.H.-F., E.K.P., E.G., C.D., and R.S.; Writing – Original Draft, P.A.T.; Writing – Review & Editing, M.S. and C.F.; Supervision, C.F. and J.R.G.; Funding Acquisition, C.F. and J.R.G.

ACKNOWLEDGMENTS

We thank Joff Silberg (Rice University, US) and Paola Costantini (University of Padova, Italy) for providing us the mito-Venus-GRX2 construct, Sue Penrhyn-Lowe (MRC Cancer Unit, Cambridge) for the support with the microscopy, and Chiara Cossetti (Cambridge Institute for Medical Research) for the support with FACS analysis. C.F., E.G., and M.S. are funded by the Medical Research Council, Core Fund to the MRC Cancer Unit, SKAG106. E.G. is funded by an MRC PhD studentship. J.R.G. and P.A.T. acknowledge the support of the University of Cambridge, Cancer Research UK (C14303/A17197) and Hutchison Whampoa Limited. P.A.T. was also funded by MRC grant 1495954. H.R.B. and J.H. are supported by the Medical Research Council (U105663141). M.E.Y. acknowledges PGS-D3 funding from the Natural Sciences and Engineering Council of Canada (NSERC) grant number PGS-D3-438137. R.S.'s work was funded by European Community's 7th Framework Programme under grant agreement FP7-PEOPLE-2013-IIF No. 628575.

Received: September 15, 2016
Revised: August 15, 2017
Accepted: September 26, 2017
Published: October 24, 2017

REFERENCES

Adam, J., Hatipoglu, E., O'Flaherty, L., Ternette, N., Sahgal, N., Lockstone, H., Baban, D., Nye, E., Stamp, G.W., Wolhuter, K., et al. (2011). Renal cyst formation in Fh1-deficient mice is independent of the Hif/Phd pathway: roles for fumarate in KEAP1 succination and Nrf2 signaling. Cancer Cell *20*, 524–537.

Akoka, S., Barantin, L., and Trierweiler, M. (1999). Concentration Measurement by Proton NMR Using the ERETIC Method. Anal. Chem. *71*, 2554–2557.

Alderson, N.L., Wang, Y., Blatnik, M., Frizzell, N., Walla, M.D., Lyons, T.J., Alt, N., Carson, J.A., Nagai, R., Thorpe, S.R., and Baynes, J.W. (2006). S-(2-Succinyl)cysteine: a novel chemical modification of tissue proteins by a Krebs cycle intermediate. Arch. Biochem. Biophys. *450*, 1–8.

Bardella, C., El-Bahrawy, M., Frizzell, N., Adam, J., Ternette, N., Hatipoglu, E., Howarth, K., O'Flaherty, L., Roberts, I., Turner, G., et al. (2011). Aberrant succination of proteins in fumarate hydratase-deficient mice and HLRCC patients is a robust biomarker of mutation status. J. Pathol. *225*, 4–11.

Baysal, B.E., Ferrell, R.E., Willett-Brozick, J.E., Lawrence, E.C., Myssiorek, D., Bosch, A., van der Mey, A., Taschner, P.E., Rubinstein, W.S., Myers, E.N., et al. (2000). Mutations in SDHD, a mitochondrial complex II gene, in hereditary paraganglioma. Science *287*, 848–851.

Blewett, M.M., Xie, J., Zaro, B.W., Backus, K.M., Altman, A., Teijaro, J.R., and Cravatt, B.F. (2016). Chemical proteomic map of dimethyl fumarate-sensitive cysteines in primary human T cells. Sci. Signal. *9*, rs10.

Bridges, H.R., Jones, A.J., Pollak, M.N., and Hirst, J. (2014). Effects of metformin and other biguanides on oxidative phosphorylation in mitochondria. Biochem. J. *462*, 475–487.

Dang, L., White, D.W., Gross, S., Bennett, B.D., Bittinger, M.A., Driggers, E.M., Fantin, V.R., Jang, H.G., Jin, S., Keenan, M.C., et al. (2009). Cancer-associated IDH1 mutations produce 2-hydroxyglutarate. Nature *462*, 739–744.

Frezza, C., Zheng, L., Folger, O., Rajagopalan, K.N., MacKenzie, E.D., Jerby, L., Micaroni, M., Chaneton, B., Adam, J., Hedley, A., et al. (2011). Haem oxygenase is synthetically lethal with the tumour suppressor fumarate hydratase. Nature *477*, 225–228.

Grivennikova, V.G., Gavrikova, E.V., Timoshin, A.A., and Vinogradov, A.D. (1993). Fumarate reductase activity of bovine heart succinate-ubiquinone reductase. New assay system and overall properties of the reaction. Biochim. Biophys. Acta *1140*, 282–292.

Hoff, K.G., Culler, S.J., Nguyen, P.Q., McGuire, R.M., Silberg, J.J., and Smolke, C.D. (2009). In vivo fluorescent detection of Fe-S clusters coordinated by human GRX2. Chem. Biol. *16*, 1299–1308.

Isaacs, J.S., Jung, Y.J., Mole, D.R., Lee, S., Torres-Cabala, C., Chung, Y.L., Merino, M., Trepel, J., Zbar, B., Toro, J., et al. (2005). HIF overexpression correlates with biallelic loss of fumarate hydratase in renal cancer: novel role of fumarate in regulation of HIF stability. Cancer Cell *8*, 143–153.

Kim, N., Ripple, M.O., and Springett, R. (2012). Measurement of the mitochondrial membrane potential and pH gradient from the redox poise of the hemes of the bc1 complex. Biophys. J. *102*, 1194–1203.

Losman, J.A., Looper, R.E., Koivunen, P., Lee, S., Schneider, R.K., McMahon, C., Cowley, G.S., Root, D.E., Ebert, B.L., and Kaelin, W.G., Jr. (2013). (R)-2-hydroxyglutarate is sufficient to promote leukemogenesis and its effects are reversible. Science *339*, 1621–1625.

McAlister, G.C., Huttlin, E.L., Haas, W., Ting, L., Jedrychowski, M.P., Rogers, J.C., Kuhn, K., Pike, I., Grothe, R.A., Blethrow, J.D., and Gygi, S.P. (2012). Increasing the multiplexing capacity of TMTs using reporter ion isotopologues with isobaric masses. Anal. Chem. *84*, 7469–7478.

Mullen, A.R., Wheaton, W.W., Jin, E.S., Chen, P.H., Sullivan, L.B., Cheng, T., Yang, Y., Linehan, W.M., Chandel, N.S., and DeBerardinis, R.J. (2011). Reductive carboxylation supports growth in tumour cells with defective mitochondria. Nature *481*, 385–388.

Navarro-Sastre, A., Tort, F., Stehling, O., Uzarska, M.A., Arranz, J.A., Del Toro, M., Labayru, M.T., Landa, J., Font, A., Garcia-Villoria, J., et al. (2011). A fatal mitochondrial disease is associated with defective NFU1 function in the maturation of a subset of mitochondrial Fe-S proteins. Am. J. Hum. Genet. *89*, 656–667.

Niemann, S., and Müller, U. (2000). Mutations in SDHC cause autosomal dominant paraganglioma, type 3. Nat. Genet. *26*, 268–270.

Ooi, A., Wong, J.C., Petillo, D., Roossien, D., Perrier-Trudova, V., Whitten, D., Min, B.W., Tan, M.H., Zhang, Z., Yang, X.J., et al. (2011). An antioxidant response phenotype shared between hereditary and sporadic type 2 papillary renal cell carcinoma. Cancer Cell *20*, 511–523.

Parent, A., Elduque, X., Cornu, D., Belot, L., Le Caer, J.P., Grandas, A., Toledano, M.B., and D'Autréaux, B. (2015). Mammalian frataxin directly enhances sulfur transfer of NFS1 persulfide to both ISCU and free thiols. Nat. Commun. *6*, 5686.

Ripple, M.O., Kim, N., and Springett, R. (2013). Mammalian complex I pumps 4 protons per 2 electrons at high and physiological proton motive force in living cells. J. Biol. Chem. *288*, 5374–5380.

Salabei, J.K., Gibb, A.A., and Hill, B.G. (2014). Comprehensive measurement of respiratory activity in permeabilized cells using extracellular flux analysis. Nat. Protoc. *9*, 421–438.

Sciacovelli, M., Gonçalves, E., Johnson, T.I., Zecchini, V.R., da Costa, A.S., Gaude, E., Drubbel, A.V., Theobald, S.J., Abbo, S.R., Tran, M.G., et al. (2016). Fumarate is an epigenetic modifier that elicits epithelial-to-mesenchymal transition. Nature *537*, 544–547.

Selak, M.A., Armour, S.M., MacKenzie, E.D., Boulahbel, H., Watson, D.G., Mansfield, K.D., Pan, Y., Simon, M.C., Thompson, C.B., and Gottlieb, E. (2005). Succinate links TCA cycle dysfunction to oncogenesis by inhibiting HIF-alpha prolyl hydroxylase. Cancer Cell *7*, 77–85.

Ternette, N., Yang, M., Laroyia, M., Kitagawa, M., O'Flaherty, L., Wolhulter, K., Igarashi, K., Saito, K., Kato, K., Fischer, R., et al. (2013). Inhibition of mitochondrial aconitase by succination in fumarate hydratase deficiency. Cell Rep. *3*, 689–700.

Thompson, A., Schäfer, J., Kuhn, K., Kienle, S., Schwarz, J., Schmidt, G., Neumann, T., Johnstone, R., Mohammed, A.K., and Hamon, C. (2003). Tandem mass tags: a novel quantification strategy for comparative analysis of complex protein mixtures by MS/MS. Anal. Chem. *75*, 1895–1904.

Tomlinson, I.P., Alam, N.A., Rowan, A.J., Barclay, E., Jaeger, E.E., Kelsell, D., Leigh, I., Gorman, P., Lamlum, H., Rahman, S., et al.; Multiple Leiomyoma Consortium (2002). Germline mutations in FH predispose to dominantly inherited uterine fibroids, skin leiomyomata and papillary renal cell cancer. Nat. Genet. *30*, 406–410.

Tyanova, S., Temu, T., Sinitcyn, P., Carlson, A., Hein, M.Y., Geiger, T., Mann, M., and Cox, J. (2016). The Perseus computational platform for comprehensive analysis of (prote)omics data. Nat. Methods *13*, 731–740.

Wittig, I., Karas, M., and Schägger, H. (2007). High resolution clear native electrophoresis for in-gel functional assays and fluorescence studies of membrane protein complexes. Mol. Cell. Proteomics *6*, 1215–1225.

Yang, Y., Valera, V.A., Padilla-Nash, H.M., Sourbier, C., Vocke, C.D., Vira, M.A., Abu-Asab, M.S., Bratslavsky, G., Tsokos, M., Merino, M.J., et al. (2010). UOK 262 cell line, fumarate hydratase deficient (FH^{-}/FH^{-}) hereditary leiomyomatosis renal cell carcinoma: in vitro and in vivo model of an aberrant energy metabolic pathway in human cancer. Cancer Genet. Cytogenet. *196*, 45–55.

Yang, Y., Valera, V., Sourbier, C., Vocke, C.D., Wei, M., Pike, L., Huang, Y., Merino, M.A., Bratslavsky, G., Wu, M., et al. (2012). A novel fumarate hydratase-deficient HLRCC kidney cancer cell line, UOK268: a model of the Warburg effect in cancer. Cancer Genet. *205*, 377–390.

Yang, M., Ternette, N., Su, H., Dabiri, R., Kessler, B.M., Adam, J., Teh, B.T., and Pollard, P.J. (2014). The Succinated Proteome of FH-Mutant Tumours. Metabolites *4*, 640–654.

Zheng, L., MacKenzie, E.D., Karim, S.A., Hedley, A., Blyth, K., Kalna, G., Watson, D.G., Szlosarek, P., Frezza, C., and Gottlieb, E. (2013). Reversed argininosuccinate lyase activity in fumarate hydratase-deficient cancer cells. Cancer Metab. *1*, 12.

Cell Reports, Volume *21*

Supplemental Information

Fumarate Hydratase Loss Causes

Combined Respiratory Chain Defects

Petros A. Tyrakis, Marie E. Yurkovich, Marco Sciacovelli, Evangelia K. Papachristou, Hannah R. Bridges, Edoardo Gaude, Alexander Schreiner, Clive D'Santos, Judy Hirst, Juan Hernandez-Fernaud, Roger Springett, John R. Griffiths, and Christian Frezza

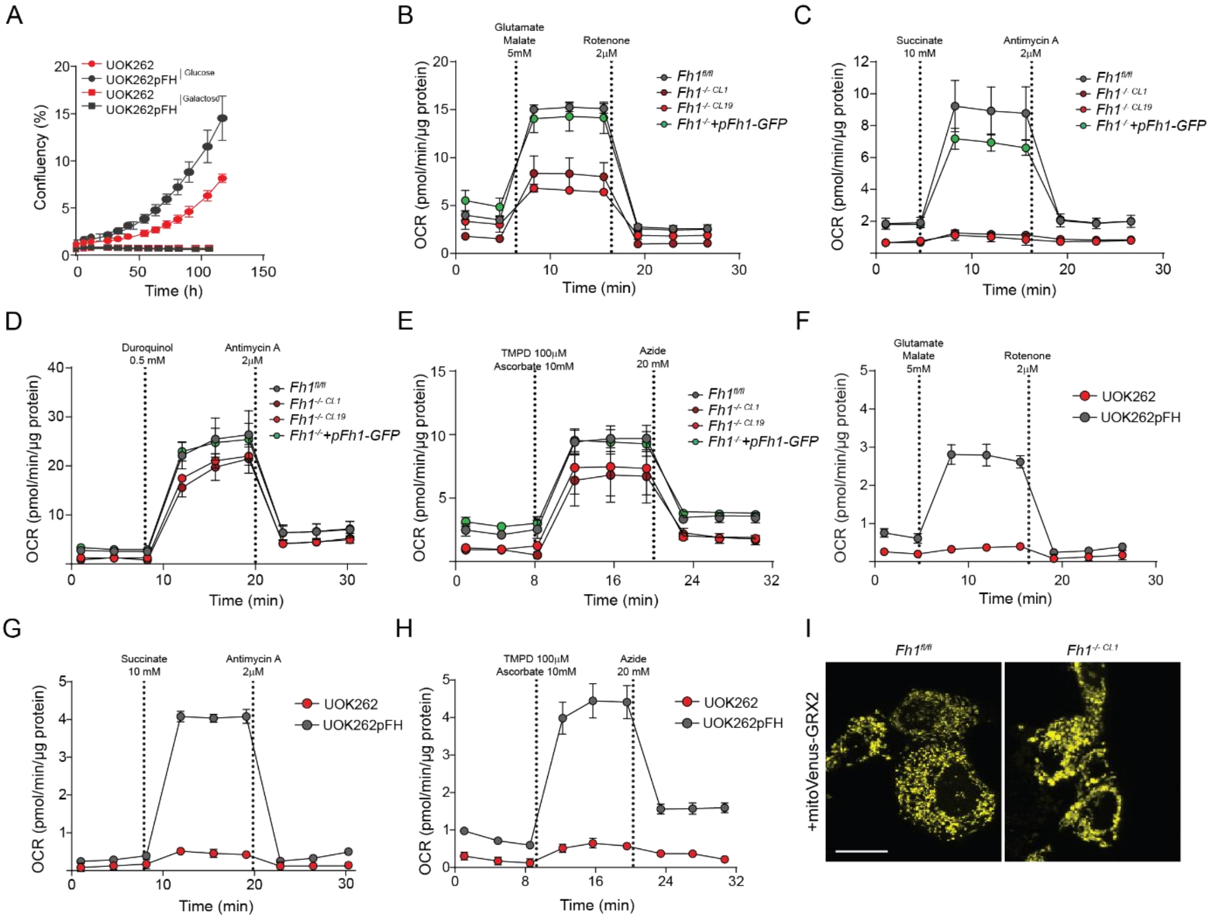

Figure S1: FH-deficient human cells display decreased respiration (related to Figure 1).

(**A**) Growth of indicated cells in 25 mM galactose (squares) or glucose (circles). Data are represented as mean ± SD. (**B-E**) Complete respiration profile of the data presented in Figure 1 and relative to mouse Fh1-deficient cells permeabilized with PMP and in the presence of substrates specific for complex I (**B**), II (**C**), III (**D**) and IV (**E**). Values are normalised for total protein content. **(F-H**) complex I (**F**), complex II (**G**), complex IV (**H**)-driven respiration in the indicated cell lines. Substrates to drive respiration and inhibitors are indicated in the graphs. Concentrations of these compounds are indicated in both the graphs and the methods section. Data were generated from at least 3 independent experiments and presented as average ± SEM**.** For respiration through CIII and CIV cells were pre-treated with Rotenone (2 µM) alone or in combination with Antimycin (2 µM) respectively before the addition of the substrates. Duroquinol was not added to the background wells used for normalisation. (**I**) Representative confocal images of cells transfected with mito-Venus-GRX2. Bar = 11.65 µm

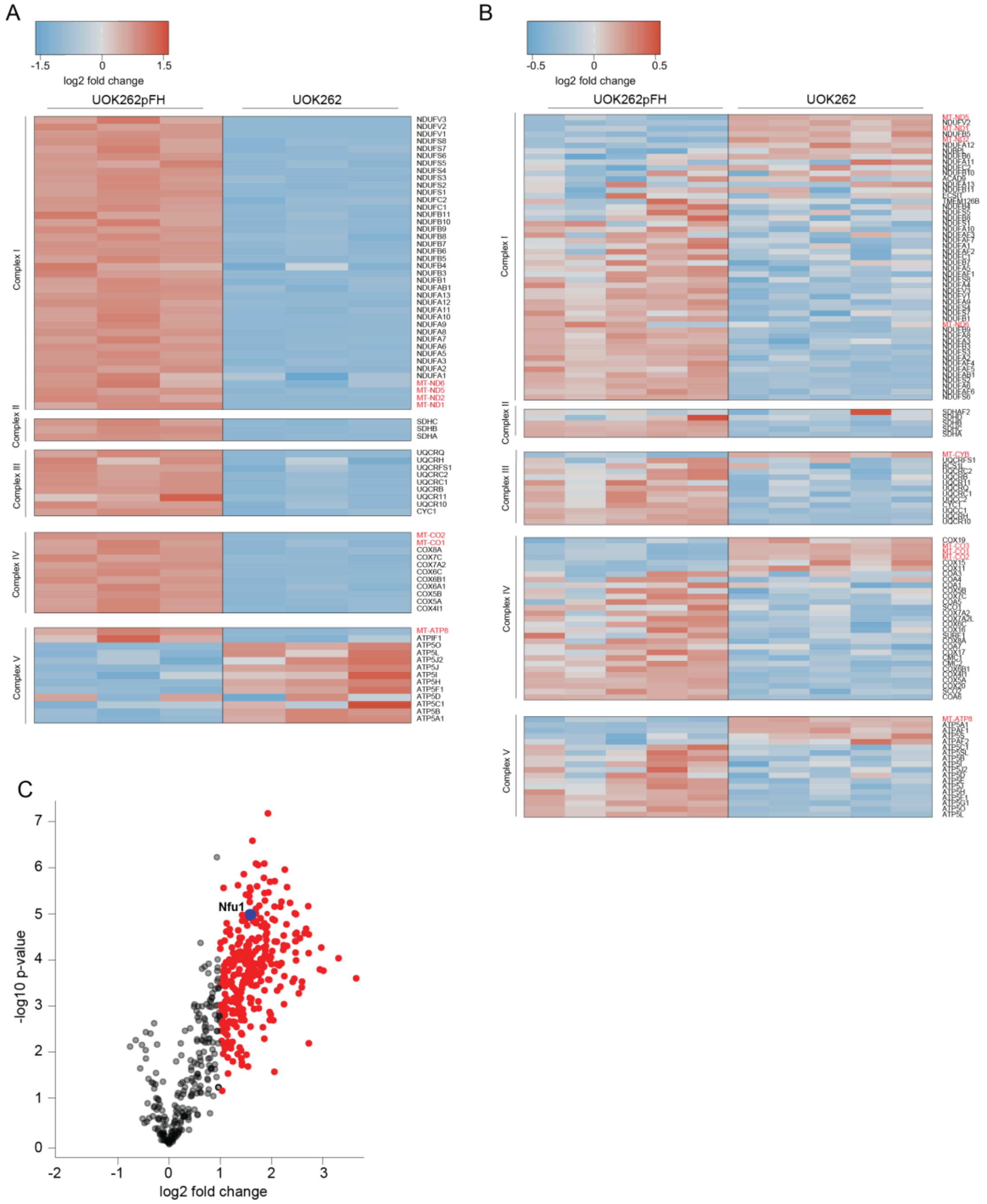

Figure S2: Abundance of respiratory chain complex subunits and assembly factors in FH-deficient cells (related to Figure 3).

(**A**) Heatmap of proteins of respiratory chain complex subunits and assembly factors in UOK262 and UOK262pFH cells. The detected subunits and assembly factors are grouped by complex. The relative quantification of N=3 biological replicates is presented. mtDNA-encoded proteins are indicated in red. (**B**) Heatmap of mRNA levels for all RC subunits from RNA-seq data in UOK262 vs UOK262pFH. mtDNA-encoded genes are indicated in red. (**C**) Volcano plot of the succinated peptides identified in UOK262 cells compared to UOK262pFH. Data were obtained from N=3 biological replicates.

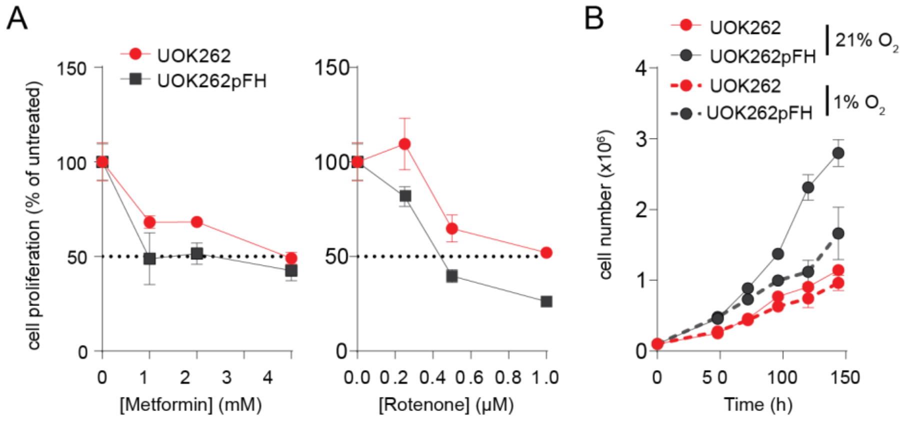

Figure S3: FH-deficient cells are resistant to RC inhibition and hypoxia (related to Figure 5).

(**A-C**) Proliferation rate of the indicated cell lines in the presence of various ETC inhibitors (**A**), and in 21% and 1% oxygen conditions (**B**). Data were obtained from at least 2 independent experiments and are presented as mean ± SD**.**
